# Supplementary material for: Identification of photoperiod-induced specific miRNAs in the adrenal glands of Sunite sheep (Ovis aries)
Source: Front Vet Sci. 2022 Jul 22;9:888207. doi: 10.3389/fvets.2022.888207 (PMC9354845; doi:10.3389/fvets.2022.888207)
Supplement: Supplementary file 1 [file Data_Sheet_1.ZIP › Supplementary file/miRDeep2 file.zip!/miRDeep2 file/Know_Structure/SP42A2/oar-mir-29b-2.pdf]

miRBase precursor : oar-mir-29b-2
Total read count : 14311
oar-miR-29b read count : 13332
remaining reads : 979

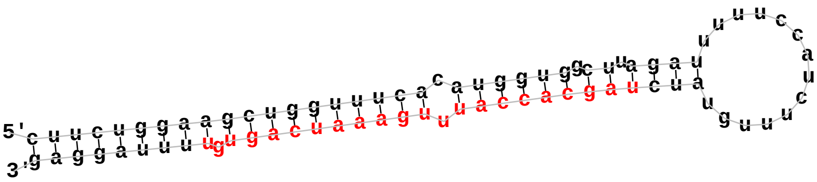

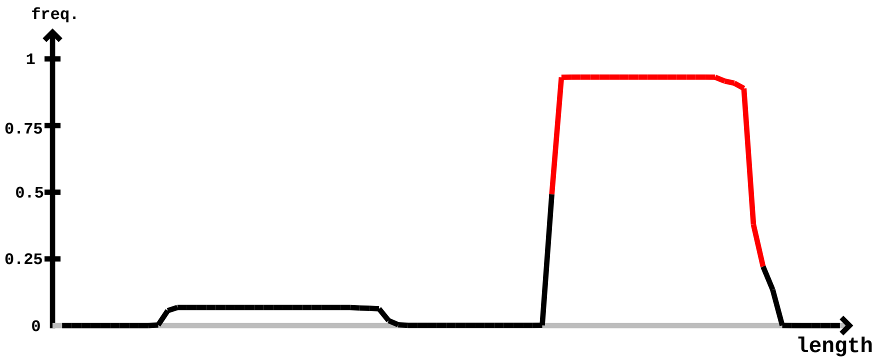

oar-miR-29b

```
5'- cuucuggaagcugguuucacaugguggcuuagauuuuuccaucuuuguaucuagcaccauuugaaaucaguguuuuaggag  -3'   exp
    (((((((((((((((((((.((((((.((.((((..............)))))))))))).)))))))))).)))))))))   reads   mm   sample
    .........gcugguuucaUauggugg......................................................   1       1    seq
    .........gcugguuucacaugguggcuu...................................................   7       0    seq
    .........gcugguuucacaugguggcuua..................................................   5       0    seq
    .........gcugguuucacaugguggcuuag.................................................   1       0    seq
    .........gcugguuucacaugguggcuuaga................................................   16      0    seq
    ..........cugguuucacaugguggcuu...................................................   20      0    seq
    ..........cugguuucacaugguggcuua..................................................   10      0    seq
    ..........cugguuucacaugguggcuuag.................................................   10      0    seq
    ..........cugguuucacaugguggcuuaga................................................   549     0    seq
    ..........cugguuucacaugguggcuuagau...............................................   162     0    seq
    ..........cugguuucacaugguggcuuagauu..............................................   20      0    seq
    ...........ugguuucacaugguggcuu...................................................   1       0    seq
    ...........ugguuucacaugguggcuuag.................................................   15      0    seq
    ...........ugguuucacaugguggcuuaga................................................   77      0    seq
    ...........ugguuucacaugguggcuuagau...............................................   59      0    seq
    ...........ugguuucacaugguggcuuagauu..............................................   17      0    seq
    ............gguuucacaugguggcuuaga................................................   1       0    seq
    .................................uuuuuccaucuuuguauc..............................   7       0    seq
    .................................uuuuuccaucuuuguauU..............................   1       1    seq
    .................................................ucuagcaccauuugaaau..............   1       0    seq
    .................................................ucuagcaccauuugaaauc.............   1       0    seq
    .................................................ucuagcaccauuugaaaucagu..........   3       0    seq
    ..................................................cuagcaccauuugaaau..............   1       0    seq
    ..................................................cuagcaccauuugaaaC..............   1       1    seq
    ..................................................cuagAaccauuugaaau..............   1       1    seq
    ..................................................cuagcaccauuugaaauc.............   88      0    seq
    ..................................................cuaCcaccauuugaaauc.............   1       1    seq
    ..................................................cuagcaccauuugaaaucG............   1       1    seq
    ..................................................cuagcaccauuugaaauca............   31      0    seq
    ..................................................cuagcaccauuugaaaucGg...........   2       1    seq
    ..................................................cuagcaccauuugaaaucag...........   62      0    seq
    ..................................................cuagcaccauuugaaaucagu..........   4197    0    seq
    ..................................................cuagcaccauuugaaaucGgu..........   33      1    seq
    ..................................................cuagcaccauuugaaaucagug.........   1705    0    seq
    ..................................................cuagcaccauuugaaaucagugu........   909     0    seq
```

oar-miR-29b

cuucuggaagcugguuucacaugguggcuuagauuuuuccaucuuuguaucuagcaccauuugaaaucaguguuuuaggag			
....cuagcaccauuugaaaucaguguu.......	4	0	seq
....uagcaccauuugUaauc.............	1	1	seq
....uagcaccauuugaaauc.............	104	0	seq
....uagcaccauuugaaauU.............	11	1	seq
....uaUcaccauuugaaauc.............	1	1	seq
....uagcaccauuugaaauA.............	1	1	seq
....uagcaccauuugaaaucG............	45	1	seq
....uagcaccauuugaaauca............	35	0	seq
....uagcaccauuugaaaucag...........	62	0	seq
....uagcaccauuugaaaucGg...........	154	1	seq
....uagcaccauuugaaaucGgu..........	868	1	seq
....uagcaccauuugaaaucagu..........	2207	0	seq
....uagcaccauuugaaaucagug.........	547	0	seq
....uagcaccauuugaaaucagugu........	329	0	seq
....uagcaccauuugaaaucaguguu.......	1910	0	seq
....uagcaccauuugaaaucaguguuu......	5	0	seq
....uagcaccauuugaaaucaguguuuu.....	1	0	seq
....agcaccauuugaaaucag...........	1	0	seq
....agcaccauuugaaaucagu..........	1	0	seq
....agcaccauuugaaaucGgu..........	1	1	seq
....agcaccauuugaaaucaguguu.......	6	0	seq
....gcaccauuugaaaucaguguu.......	1	0	seq
